# Schizophrenia and Glutathione: A Challenging Story

**DOI:** 10.3390/jpm13111526

**Published:** 2023-10-25

**Authors:** Barbara Carletti, Nerisa Banaj, Fabrizio Piras, Paola Bossù

**Affiliations:** 1 Laboratory of Neuropsychiatry, Clinical Neuroscience and Neurorehabilitation Department, IRCCS Santa Lucia Foundation, Via Ardeatina 306, 00179 Rome, Italy; n.banaj@hsantalucia.it (N.B.); f.piras@hsantalucia.it (F.P.); 2 Laboratory of Experimental Neuropsychobiology, Clinical Neuroscience and Neurorehabilitation Department, IRCCS Santa Lucia Foundation, Via del Fosso di Fiorano 64, 00143 Rome, Italy; p.bossu@hsantalucia.it

**Keywords:** schizophrenia, glutathione, oxidative stress, neurodegeneration, biomarker

## Abstract

Schizophrenia (SZ) is a devastating mental illness with a complex and heterogeneous clinical state. Several conditions like symptoms, stage and severity of the disease are only some of the variables that have to be considered to define the disorder and its phenotypes. SZ pathophysiology is still unclear, and the diagnosis is currently relegated to the analysis of clinical symptoms; therefore, the search for biomarkers with diagnostic relevance is a major challenge in the field, especially in the era of personalized medicine. Though the mechanisms implicated in SZ are not fully understood, some processes are beginning to be elucidated. Oxidative stress, and in particular glutathione (GSH) dysregulation, has been demonstrated to play a crucial role in SZ pathophysiology. In fact, glutathione is a leading actor of oxidative-stress-mediated damage in SZ and appears to reflect the heterogeneity of the disease. The literature reports differing results regarding the levels of glutathione in SZ patients. However, each GSH state may be a sign of specific symptoms or groups of symptoms, candidating glutathione as a biomarker useful for discriminating SZ phenotypes. Here, we summarize the literature about the levels of glutathione in SZ and analyze the role of this molecule and its potential use as a biomarker.

## 1. Introduction

Schizophrenia is one of the most serious and debilitating psychiatric illnesses [1,2,3], characterized by three groups of symptoms: positive, negative and cognitive symptoms. Positive symptoms consist of hallucinations (perceptions in the absence of an external stimulus) and delusions (fixed and false beliefs arising internally). Negative symptoms include decreased emotional expression, amotivation, apathy and social withdrawal. Impaired cognitive functions involve disorganization of thought, deficit in long-term memory and sustained attention, problems in processing speed [4]. The psychiatric diagnosis of SZ remains centered on the analysis of clinical symptoms, as the pathophysiology of the disorder is still unclear, and there is a lack of appropriate biomarkers. Many alternative hypotheses have been formulated regarding the pathologic mechanisms of SZ, whose developmental and neurodegenerative nature is still a matter of debate [5]. The pathogenic pathways include, among others: impaired neurotransmission, neuroinflammation, autoimmune dysfunctions, oxidative stress, defects of myelination [6]. Although the suggested mechanisms are multiple and disparate, the general idea is that they may converge on a common pathway [7]. One of the candidates for this process is oxidative stress [8]. Several studies suggested a role for oxidative stress in the molecular mechanisms involved in the disease etiology. Evidence for oxidative damage, including decreased levels of antioxidants and in particular reduced glutathione (GSHr) and total glutathione (GSHt, that is the sum of GSHr and its oxidized form GSSG), has been found in body fluids and postmortem brain tissues of patients affected by SZ [9,10,11]. The present review focuses on the antioxidant glutathione, revisiting the literature on its role in the disease and discussing the possibility of considering this molecule as a novel biomarker for SZ. Notably, since schizophrenia is a complex and heterogeneous disorder, GSH could be a promising biomarker to identify different SZ phenotypes.

## 2. Oxidative Stress and Glutathione

Oxidative stress is caused by an imbalance between the excessive production of reactive oxygen species (ROS) and the cellular antioxidant defense, which is usually able to counteract the reactive compounds and repair the resulting injury [12]. The ROS production occurs inside the cell during normal aerobic metabolism and mainly for mitochondrial respiration with its consequent incomplete reduction in oxygen to water. ROS include both free radicals (containing highly reactive unpaired electron), such as: superoxide (O_2_-^.^) and hydroxyl radical (OH^.^), and other molecular species, like hydrogen peroxide (H_2_O_2_). Prolonged exposure to these oxidant intermediates determines the oxidation of cellular components (proteins, lipids and DNA). Under normal physiologic conditions, the cell deals with the flux of ROS. Oxidative stress occurs when the ROS level exceeds the antioxidant systems. The brain is a sensitive site of oxidative damage due to its high metabolic rate such that consuming a large amount of inspired oxygen produces many reactive species [13]. In addition, the brain has a low level in antioxidant systems and a reduced capacity for cellular regeneration that worsen its condition. The most prevalent antioxidant in the brain is glutathione, a tripeptide made up of cysteine, glutamate and glycine. It is essential for cellular detoxification of reactive oxygen species in the central nervous system, and oxidative stress together with an altered glutathione metabolism may be implicated in the axonal degeneration observed in various neurodegenerative diseases [14]. Glutathione exists in a reduced state (GSHr) able to scavenge free radicals and in an oxidized state (GSSG) capable of causing protein S-glutathionylation, a process that consists in the connection of mixed disulphide bonds between glutathione and protein cysteine residues (Cys). In GSH metabolism, the oxidation of two molecules of GSHr generates one molecule of GSSG that, in turn, can be reduced back by the enzyme GSH reductase. The ratio between GSHr and GSSG has the function of maintaining the redox balance and potential in the cell, and in normal condition, it is about 100. Under oxidative stress, the GSHr/GSSG ratio decreases to 10 or less, and these values are able to trigger protein S-glutathionylation, which, in turn, can alter the function, interactions and localization of proteins. The oxidation of Cys residues by ROS can cause irreversible modifications that bring to dangerous dysfunction, thus GSH protects the proteins by reducing their thiols (i.e., their –SH functional groups). More importantly, abnormal protein S-glutathionylation has been considered the cause of several neurodegenerative diseases exacerbating the injury of oxidative stress. The brain level of GSH is about 2–3 mM. Interestingly, its distribution is highest in the cortex and progressively diminishes in the cerebellum, hippocampus, striatum and substantia nigra [15]. The growing evidence of the different expression of glutathione in the diverse cerebral regions has suggested the idea that the GSH level paralleled a different vulnerability of the relative brain areas to oxidative stress and, as a consequence, to neurodegeneration that could be part of the cause of SZ [15,16]. Despite the various hypotheses that could explain the role of GSH in the pathophysiology of SZ, it is still unclear whether the glutathione-mediated oxidative damage represents the cause or the consequence of the SZ impairment. An intriguing idea is that a possible cause of oxidative stress is linked to mitochondrial dysfunction. Neurons strictly depend on mitochondria to execute diverse physiological processes. A comprehensive analysis of the potential interactions between mitochondrial function and neuronal activity is important to understand the pathophysiology of schizophrenia. Mitochondrial dysfunction may lead to impaired neuronal functions through a variety of mechanisms linked to oxidative stress, including calcium homeostasis disruption, the alterations in excitatory signaling related to NMDAR and PV interneurons malfunction, all processes involved in SZ pathophysiology. From the literature, it emerges that the role of GSH in SZ is ambiguous, and its possible explanation will be described in the following paragraphs.

## 3. Glutathione in Schizophrenia

Growing evidence suggests that the dysregulation of GSH metabolism may have a crucial part in SZ pathophysiology. A huge number of studies report a reduction in GSHr in tissues of SZ patients compared to controls. In GSH metabolism, the GSHr/GSSG ratio and the GSH levels are the primary causes of oxidative balance. It is, in other words, fundamental for the cell to maintain a high GSHr level, a low GSSG level and appropriate GSHt values. In SZ conditions, decreased levels of GSHr and GSHt (generally indicated as relative measures, referred to as either controls or average levels) have been reported in blood, including plasma [11,17,18,19,20,21,22,23], serum [24,25,26,27,28,29,30] and erythrocytes [31,32,33,34], but also in cerebrospinal fluid (CSF) and postmortem brain tissue of patients [9,10,35].

However, there are also data not consistent with the above mentioned results. For example, in the work of Samuelsson and colleagues [36], no differences in plasma and CSF levels of GSH were observed in SZ patients, as compared to controls. Langbein et al. [37] found that patients with first-episode psychosis (FEP) had normal erythrocyte levels of GSHr, GSHt and GSSG. The study of Martinez-Cengotitabengoa [38] also showed no changes in erythrocyte levels of GSHt in SZ patients, as compared to control subjects.

Part of the variability might be due to the different analytical methods and the differences in the processing and/or storage of the biological samples. For example, red blood cells are responsible for the major amount of GSH in the blood, and a slight hemolysis (0.1%–1%) can result in an erroneous high value of GSH in plasma [39,40]. Another complication is the oxidation of GSH that occurs in the plasma within 5 min at room temperature, even in the presence of a preservative solution, causing a sensitive loss of the molecule recovery [39]. Additional variabilities could be due to differences in the selection of the sample population among studies, because of different exclusion criteria for patients’ selection or stage and severity of the disease. However, no evidence has been found to support the hypothesis that dietary antioxidant intake and smoking can contribute to GSH dysregulation in SZ [41]. Concerning the antipsychotic medications, a recent review found that there are still no sufficient and accurate studies able to confirm that they induce an alteration in GSH levels [42].

It is also of particular interest to determine whether the GSHr/GSSG ratio, GSHr, GSHt or GSSG levels can correlate to stages, severity and type of symptoms [43]. Many authors suggested that GSH levels may vary according to disease stage (prodromic phase, first episode of psychosis and chronic condition), but this aspect would necessitate further analysis. Nonetheless, the literature reported studies on the diverse phases of the illness, though not all in parallel. Alongside the studies already mentioned and preponderantly obtained in chronic patients, there are works specifically regarding the prodromic stage and first episode of psychosis. Accordingly, a crucial work of Lavoie and colleagues [34] showed that a GSH decrease in erythrocytes is a good predictor for the transition to psychosis in a population at high risk. Since individuals at high risk of psychosis do not necessarily develop the illness, it is crucial to find a biomarker able to distinguish between subjects who transit to psychosis and those who do not. The article highlights that “low erythrocyte GSH” is a good predictor for the onset of disease. Nevertheless, the work of Da Silva et al. [44] using 3T proton magnetic resonance spectroscopy reported no significant difference in glutathione levels of medial prefrontal cortex (mPFC) in antipsychotic naïve individuals, who are at clinically high risk of developing psychosis, as compared to healthy volunteers, suggesting that an alteration in glutathione metabolism does not predate the onset of psychosis, at least in the described conditions.

In first-episode psychosis, markers of oxidative damage have been demonstrated to be associated with positive [33] and negative symptoms [45,46] as well as cognitive impairment [38]. More precisely, total GSH and GSHr were decreased, while GSSG increased in FEP [21,29,31,47], indicating that glutathione may be an early indicator of oxidative stress in the course of schizophrenia. Of particular interest is the correlation between GSSG increase and cognitive impairment, which is a common clinical feature of the disease and could thus be due to oxidative-stress-mediated neurodegeneration [48]. A similar hypothesis has been formulated also in the work of Fraguas and colleagues [49], which investigated the GSH levels and their correlation with cortical grey matter (GM) volume. The latter study confirmed that a decrease in GSH is related to GM loss, suggesting a role for glutathione-mediated oxidative stress in neuronal death.

Thus, there is considerable evidence of GSH metabolism dysregulation in the different stages of the disease, although findings are not always consistent.

The GSH levels and their relation with the severity of clinical symptoms are also matters of conflicting results. Raffa et al. [18] reported an inverse correlation between the clinical global impression-severity scores and the levels of total GSH and GSHr. In accordance with these results, Tsai et al. [28] also demonstrated a significative negative association of GSH levels with SZ in the acute phase, and the study of Juchnowicz et al. [50] found that GSH could be useful in distinguishing FEP and chronic patients from controls. Overall, some studies showed that GSHr levels are associated with the severity of both positive or negative symptoms [18,22,31,38,41,51,52,53], while others do not observe any association [27,32,47,54,55,56]. Therefore, despite the contradictions observed among the different studies, the GSH involvement in SZ pathophysiology and its correlation with disease stages, phenotypes and severity of the symptoms, while still unclear, strengthens the need to address additional research efforts to investigate a possible role of this antioxidant molecule as an SZ biomarker.

## 4. From the Periphery to the Center

The association between peripheral and central GSH levels in SZ, primarily in its chronic stage, allows us to draw interesting observations. Magnetic resonance spectroscopy (MRS) studies reported a GSH decrease in some SZ-damaged brain areas, such as the anterior cingulate cortex (ACC), thalamus, striatum and medial prefrontal cortex [9,43,57,58,59]. In particular, the consistent decline of GSH levels detected in ACC is confirmed by a metanalysis revision. This result links the peripheral datum in the blood with that in the brain, indicating that peripheral glutathione levels can mirror the brain’s oxidative condition. A similar trend was also observed in the prefrontal cortex, but additional efforts are necessary to reach a relevant consistency of these data. Furthermore, the same observations have not been reported in the insula and visual cortex that are implicated in the disease [59], while one group indicated a GSH elevation in the medial temporal lobe [56]. MRS studies are particularly fascinating though ambiguous, for example: three groups reported no change in the posterior medial prefrontal cortex [52] and anterior cingulate cortex [60], as well as in the medial prefrontal lobe [61]. It was also revealed that a higher brain level of GSH is accompanied by a faster re-sponse to antipsychotic treatment in drug-naïve FEP [62], and a study by Wang et al. [43] confirmed a sensible reduction in GSHr in ACC and thalamus (but not in other brain regions like the cortex semioval, orbitofrontal cortex, and dorsolateral prefrontal cortex) in treatment-resistant patients compared to nonresistant. The first study measuring GSH level in the prefrontal cortex with MRS was performed by Do et al. [9], and its findings of a consistent reduction in GSH were corroborated by a reduction in GSH also in CSF. This approach is very important, as it strengthens MRS measurement of central GSH levels with those in the periphery (CSF and blood), and it should be always performed, whenever possible.

Many are the variables that could explain the presence of the diverse discrepancies among the MRS studies [63]. The difference in brain areas, the strength of the magnetic fields used (1.5, 3, 4 and 7T) making it difficult to discriminate the spectrum of GSH due to the overlap with other neurochemicals, and the type of acquisition strategy all may affect the final results, in addition to the criteria of patients’ selection such as: FEP vs. chronic, and drug-free vs. medicated. The group of Palaniyappan [64] suggested an alternative and interesting hypothesis, positing that the difference in GSH levels could be due, at least partially, to the presence of two subtypes of patients: one with evident GSH deficit and another one with a GSH condition rather comparable to that of controls. These subgroups may differ in disease outcome regarding parameters like disease severity or progression, as well as response to treatments. Hence, the different vulnerability of patients to oxidative stress may account for the diverse MRS results. In addition, it is reasonable to suppose that not all brain areas of interest in neuronal damage in SZ owe their condition directly to oxidative stress, but some of them may rather be affected as a consequence of the oxidative injury of connected areas.

## 5. N-Acetylcysteine: A Support to GSH Implication in SZ

The implication of GSH in SZ is also supported by preclinical and clinical studies conducted with the antioxidant drug N-acetylcysteine (NAC), which is a precursor of L-cysteine and plays a role as a cysteine donor for GSH synthesis. Preclinical studies showed that its use determined an increase in plasma cysteine levels and a subsequent involvement in rising GSH levels [65]. It has also been shown that NAC penetrates the blood–brain barrier (BBB), augmenting GSH concentration in the brain [66,67]. Works performed in SZ animal models demonstrated that the administration of NAC reverses GSH depletion and behavioral deficit [68,69]. Placebo-controlled trials confirmed that NAC treatment increases glutathione levels in the medial prefrontal cortex in early-phase SZ [70], and several clinical studies found that NAC led to a significant reduction in positive, negative and cognitive symptoms in patients who either underwent antipsychotic therapy or were untreated [71,72,73,74,75,76]. However, the results regarding negative symptoms are more prominent than those of positive symptoms. An intriguing finding is the correlation between the baseline level of GSH and the effect of NAC, as a low level appears associated with a beneficial effect of NAC supplementation mostly on positive symptoms [70,77]. In early-phase SZ, an interesting beneficial effect of NAC administration in protecting white matter integrity has also been discovered, with a six-month treatment that increases the functional connectivity along the cingulum and the fornix [78,79]. This finding collocates glutathione dysregulation in the optic of playing a role in neurodegeneration and provides additional data on the link between glutathione-mediated oxidative stress and white matter impairment in SZ.

## 6. NMDAR Hypofunction and Its Link to Glutathione in SZ

The two major hypotheses formulated to explain the pathophysiological involvement of GSH in SZ are the N-methyl-d-aspartate (NMDA) receptor (NMDAR) hypofunction, or glutamatergic hypothesis, and the myelination impairment. It has been demonstrated that NMDAR hypofunction acts synergistically with oxidative stress, revealing an interesting SZ pathophysiological mechanism. In adult SZ rodent model systems, both NMDAR hypofunction and oxidative stress induce similar behavioral and cognitive disturbances, suggesting that they may be reciprocally correlated [6,80]. Steullet and colleagues [81] found that GSH deficit determines a reduced NMDAR function in rat hippocampal slices. The hypothesis is that GSH depletion may lead to an alteration of the redox state and to an oxidative modification of redox-sensitive sites of NMDAR. Particularly vulnerable to NMDAR hypofunction are also parvalbumin (PV) interneurons, presumed to be implicated in SZ pathophysiology. The NMDAR contains redox-sensitive active sites located on cysteine residues, and glutathione is able to depress the function of the receptor by oxidizing those sites [82,83]. Moreover, depletion of GSH leads to NMDAR impairment of synaptic plasticity with a decrease in the response and LTP [81,84]. On the other hand, NMDAR hypofunction can reduce GSH levels, contributing to a further redox imbalance. It has also been demonstrated that NMDAR antagonists increase ROS production [85,86,87] and that synaptic activity mediated by NMDAR increases the GSH synthesis, coping with the need of antioxidant requested by neuron activity to avoid oxidative stress [88]. This scenario highlights a reciprocal NMDAR–GSH link. The glutamatergic hypothesis proposes that NMDAR hypofunction governs the damage of PV fast-spiking GABAergic interneurons and their synchronization activity [89], resulting in the diverse SZ symptoms. According to this hypothesis, the neuronal cell death revealed in hippocampal and cortical areas of SZ patients may be due to the disinhibition of glutamatergic projection neurons followed by excitotoxic damage. NMDARs are located on GABAergic inhibitory interneurons that generally are activated by the stimulation of these receptors. The NMDAR hypofunction drives the lack of activation of interneurons and the relative inhibition that these cells exert on glutamatergic projection neurons. Thus, a lack of inhibition of glutamatergic neurons leads to an increment in their neurotransmission. The excessive release of glutamate may cause a downstream cascade of excitotoxic events, ending up in neuronal cell death. Interestingly, Yang et al. [90] generated a glutamate–cysteine ligase (GCL) modifier subunit KO mice (gclm -/-) to disrupt GSH synthesis and found that this mouse model system is associated with schizophrenia [91]. GCLM-KO mice display about 70% reduction of brain GSH levels and a decrement in PV interneurons, as reported by Steullet and colleagues [92]. Considering these findings, it can be reasoned that interneuron impairment may bring to the development of different SZ phenotypes depending on the different neural circuitry involved in the damage.

Among the neurobiological anomalies derived from oxidative stress and potentially involved in the pathophysiology of SZ, there is also abnormal myelination. In fact, widespread disruption of white matter integrity has been observed in SZ. This pattern of myelination damage is typical in both GCLM-KO mice and SZ patients. The oligodendrocytes and their progenitors are highly susceptible to redox dysregulation. It is known that glutathione depletion causes the death of oligodendrocytes and their precursors [93,94], with a consequent deficit in myelination. Monin et al. [61] investigated the role of GSH in this oligodendroglia-mediated myelination damage and found that GSH levels in the medial prefrontal cortex are associated with white matter integrity. The GSH deficit correlates with oligodendrocyte- and myelin-associated protein depletion caused by a decreased proliferation of oligodendrocyte progenitor cells via Fyn kinase upregulation. The myelination of the prefrontal lobes comes to completion only in early adulthood, and studies on GCLM-KO mice evidenced a myelin deficit of the anterior cingulate cortex only in peripubertal period, suggesting the presence of a critical developmental phase for redox regulation of the process. Abnormal myelination might account for an altered conduction velocity, which, in turn, affects the synchronization of the neuronal signal. This process could underline SZ symptoms [84,95].

## 7. Glutathione as a Biomarker for Schizophrenia

The accumulating evidence of GSH involvement in SZ pathophysiology candidate this antioxidant molecule as an SZ biomarker. Given that direct access to the brain is impossible, peripheral tissues such as blood and cerebrospinal fluid are considered good sources for identifying potential biomarkers and molecular mechanisms that underlie SZ. Indeed, there is a significant and constantly growing interest in searching for blood-based biomarkers to assist in the SZ diagnosis. Blood biomarkers are easily accessible, minimally invasive and, at the same time, not expensive, therefore optimal for clinical use. Glutathione could be one of these markers, as it can cross the BBB in its reduced form [96], but a number of controversial outcomes characterize the GSH role in SZ, probably because of the complexity of this mental illness and its great heterogeneity of symptoms. In this scenario, the variability of results regarding the GSH levels in SZ patients may reflect the different disease conditions and may allocate glutathione as a biomarker for the SZ states, where we intended for SZ “state” the different groups of symptoms that define the phenotype of a subject (i.e., deficit or non-deficit schizophrenia). To define a biomarker able to distinguish the different states of a complex disease is crucial to favor a correct diagnosis and to drive the treatments in the context of personalized medicine.

As before reported and summarized in Table 1, several observations are emerging from a variety of biochemical and imaging techniques that provide a composite picture of the role of glutathione in SZ.

Indeed, a wide body of evidence shows a reduction in glutathione levels in plasma and serum of chronic and FEP SZ patients that makes into correlation the dysregulation of GSH metabolism not only with the chronic stage, but also with the initial acute phase of the pathology, suggesting that glutathione could be an early biomarker of SZ. Overall, the data corresponding to blood GSH measurement appear strong and indicate the possibility of considering glutathione as a general biomarker of SZ, whereas no substantial evidence emerges from the reported studies about its precise correlation with severity, stage or phenotype symptoms (i.e.: SZ deficit or non-deficit conditions that are characterized by negative and positive symptoms, respectively).

**Figure 1:**
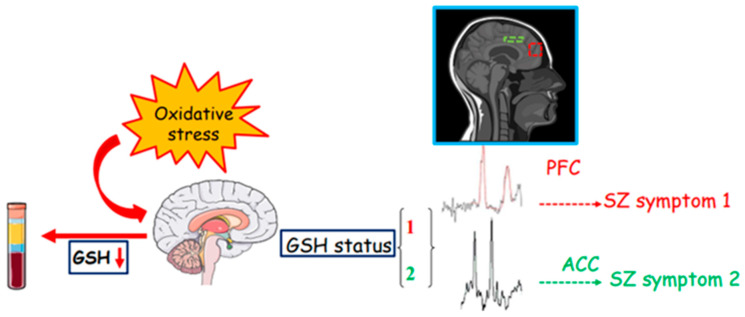
Integrative figure regarding the possibility of the diverse glutathione status to mark the different SZ conditions. Parts of this figure were drawn by using pictures from Servier Medical Art. Servier Medical Art by Servier is licensed under a Creative Commons Attribution 3.0 Unported License (https://creativecommons.org/licenses/by/3.0/). The figure of the graph number 2 is cropped from the image of DJ Manton (https://commons.wikimedia.org/wiki/File:MRS_spectrum.gif), licensed by Creative Commons Attribution 3.0 Unported.

However, the contradictory results of GSH levels among studies (especially those using MRS) could be explained by a different vulnerability of the brain regions to oxidative damage. Accordingly, a different expression of glutathione in the diverse brain areas has been shown in mouse models, where a higher GSH expression may indicate a condition less prone to oxidative stress. Different human brain regions and subregions likely show different GSH expressions, and a GSH measurement conducted without distinguishing between the diverse areas may lose these differences. Thus, the initial baseline level of glutathione accounting for the propensity to an oxidative insult in the corresponding region should be considered. Furthermore, the oxidative damage could be explained by the glutamatergic hypothesis with a PV interneuron degeneration. All the circuitry affected by the interneuron damage may contribute to the specific SZ symptoms. Accordingly, Cabungcal et al. [97] showed that glutathione deficit induces the GABAergic interneuron degeneration in the ACC of an SZ rodent model system. A systematic investigation conducted to evaluate the possible association between ACC GSH levels and the different SZ symptoms, dissecting for example the different parts of ACC and anhedonia–asociality or avolition–apathy for the negative symptoms, would be a huge challenge. The MRS analysis coupled with GSH peripheral detection could allow for distinguishing between the different SZ states as long as they are conducted in precise brain areas relevant to functions correlated to specific signs (Figure 1).

In addition, SZ symptoms are complex and should be analyzed in all their components. For instance, it is predictable that in positive symptoms, visual or hearing hallucinations involve different brain areas that are distinct from those of delusions. Therefore, a general SZ sufferance detected by peripheral GSH levels in plasma and serum, where the measurements are apparently more consistent than those in erythrocytes, could be paralleled by a more precise investigation with brain MRS analysis that, in turn, could reveal a particular SZ state (Figure 1). Overall, we believe that peripheral (i.e., CSF or blood) GSH levels may guide the diagnosis of SZ illness and that a subsequent and targeted MRS measurement could help in understanding the origin and severity of the symptoms.

To focus on the impairment of the synaptic and neurotransmission machinery offers an alternative strategy to consider glutathione as an SZ biomarker. In fact, these processes have been demonstrated to be altered in the brains of SZ patients. More precisely, a metanalysis study demonstrated that hippocampus, frontal and cingulate cortices presented a reduction in synaptophysin [98], and two studies by Thompson and colleagues [99,100] highlighted the presence of altered levels of the SNARE protein SNAP-25 in CSF of SZ patients. The hypothesis of the authors is that increased SNAP-25 in the CSF should represent an increment of exocytosis within the brain and therefore a reduction in the proteins in situ within the brain. According to this process, it would be of particular interest to investigate the peripheral concentration of specific proteins that have undergone glutathionylation and their use as potential biomarkers of synapse loss or neurodegeneration in SZ. In fact, a fascinating idea is that the imbalance of GSH metabolism may determine a dysregulation of the glutathione bound to proteins, altering their function and thus causing the damage responsible for the schizophrenic phenotypes.

## 8. Conclusions

In conclusion, even if the role of glutathione in the pathophysiology of SZ is largely recognized, the heterogeneity of results poses several problems and reflects not only the disparity of methods used and experimental systems performed among the studies but also the possible influence of the variability in SZ phenotypes, stage and severity of the disease. Therefore, we believe that this heterogeneity, rather than a mere problem of techniques, is an intriguing puzzle that could redefine the classification of SZ patients. In this scenario, the identification of glutathione as a diagnostic biomarker able to distinguish between patient subtypes leads to a promising and challenging concept, which could pave the way for innovative personalized approaches in the treatment of SZ. Here, the basic concept is that the discrepancies regarding GSH levels between studies in SZ do not prevent candidating GSH as a biomarker, but they rather reflect the diverse SZ conditions. Thus, each GSH status may show relationships with different schizophrenia-associated phenotypes, resulting in a promising biomarker for diagnosis and treatment response. However, this idea needs to be validated and requires additional specific research approaches able to investigate and recognize the relationships that link the different GSH conditions with the symptoms presented by SZ subjects.

## Figures and Tables

**Table 1:** Studies on differences in glutathione levels in schizophrenia. Abbreviations: (SZ) chronic schizophrenia, (FEP) first episode of psychosis, (PFC) prefrontal cortex, (CSF) cerebrospinal fluid, (ACC) anterior cingulate cortex, (DLPFC) dorsolateral prefrontal cortex, (OFR) orbitofrontal region, (CSO) centrum semioval, (/) no significative changes or correlation, (PANSS) positive and negative symptoms score.

Study Population	Sample	GSH Status	Note/SZ Symptoms	Reference
**SZ**	Plasma	GSHr↓		Dietrich-Mutsazalska et al. (2009) [17]
**SZ**	Plasma	GSHt, GSHr↓		Raffa et al. (2009) [18]
**SZ**	Plasma	GSHt, GSHr, GSSG↓		Raffa et al. (2012) [19]
**FEP**	Plasma	GSHr↓		Ruiz-Litago et al. (2012) [21]
**SZ**	Plasma	GSHt↓	PANSS↑	Nucifora et al. (2017) [22]
**SZ**	Plasma	GSHr↓		Guidara et al. (2020) [23]
**SZ**	plasma, lymphocytes	GSHt↓		Couglin et al. (2021) [11]
**SZ**	Plasma	GSHt (/)		Samuelsson et al. (2013) [36]
**SZ**	Serum	GSHr↓		Ivanova et al. (2015) [24]
**SZ**	Serum	GSHr↓		Fukushima et al. (2014) [26]
**SZ**	Serum	GSHr↓	PANSS (/)	Gonzalez-Liencres et al. (2014) [27]
**SZ**	Serum	GSHr↓	PANSS↑	Tsai et al. (2013) [28]
**FEP**	Serum	GSSG↑	PANSS (/)	Tao et al. (2020) [29]
**SZ**	Serum	GSHr↓		Cruz et al. (2021) [30]
**SZ**	Erythrocytes	GSHr↓		Altuntas et al. (2000) [32]
**Risk of psychosis**	Erythrocytes	GSHr↓		Lavoie et al. (2017) [34]
**FEP**	Erythrocytes	GSHt↓, GSHr↓, GSSG↑	GSHt, GSHr positive correlation with positive symptoms	Raffa et al. (2011) [31]
**FEP**	Erythrocytes	GSHt (/), GSHr (/), GSSG (/)		Langbein et al. (2018) [37]
**FEP**	Erythrocytes	GSHt↓		Mico et al. (2011) [47]
**FEP**	Erythrocytes	GSHt↓		Fraguas et al. (2012) [49]
**FEP**	Erythrocytes	GSHt (/)		Martinez-Cengotitabengoa et al. (2012) [38]
**FEP**	erythrocytes	GSHr↓		Martinez-Cengotitabengoa et al. (2014) [55]
**SZ**	erythrocytes	GSHr↓		Dadheech et al. (2012) [54]
**SZ**	Prefrontal cortex	GSHt, GSHr, GSSG↓		Gawryluk et al. (2011) [10]
**SZ**	caudate	GSHr↓, GSSG↓, GSHr:GSSG↓		Yao et al. (2006) [35]
**SZ**	CSF	GSHr↓		Do et al. (2000) [9]
**SZ**	Whole blood	GSHr↓ GSSG↑		Ballesteros et al. (2013) [41,51]
**FEP**	Striatum	GSHr↓	PANSS↓ positive correlation	Reyes-Madrigal et al. (2019) [57]
**FEP**	ACC, Thalamus DLPFC OFR CSO	GSHr↓ GSHr↓ GSHr (/) GSHr (/) GSHr (/)	correlation with severity of cognitive symptoms	Wang et al. (2019) [43]
**SZ**	Prefrontal cortex	GSHr↓		Do et al. (2000) [9]
**SZ**	ACC Insula visual cortex	GSHr↓ GSHr (/) GSHr(/)		Kumar et al. (2020) [59]
**FEP**	Medial temporal lobe	GSHr↑		Wood et al. (2009) [56]
**SZ**	Posterior medial frontal cortex	GSHr (/)		Matsuzawa et al. (2008) [52]
**SZ**	Medial prefrontal cortex	GSHr (/)		Monin et al. (2015) [61]
**SZ**	ACC	GSHr (/)		Terpstra et al. (2005) [60]
**Risk of psychosis**	mPFC	GSHr (/)		Da Silva et al. (2018) [44]
**FEP**	Dorsal ACC	GSHr (/)		Dempster et al. (2020) [62]

## Data Availability

Not applicable.
